# Low Dose Epigallocatechin Gallate Alleviates Experimental Colitis by Subduing Inflammatory Cells and Cytokines, and Improving Intestinal Permeability

**DOI:** 10.3390/nu11081743

**Published:** 2019-07-29

**Authors:** Yong Du, Huihua Ding, Kamala Vanarsa, Sanam Soomro, Sahar Baig, John Hicks, Chandra Mohan

**Affiliations:** 1 Department of Biomedical Engineering, University of Houston, Houston, TX 77204, USA; 2 Department of Pathology, Texas Children’s Hospital, Houston, TX 77030, USA

**Keywords:** inflammatory bowel disease, epigallocatechin gallate (EGCG), cytokines, animal model

## Abstract

Background: In this study, we investigate the impact of epigallocatechin gallate (EGCG), the most abundant and potent catechin in green tea, on a mouse model of inflammatory bowel disease (IBD) and the underlying mechanisms of action. Methods: C57BL/6J mice were subjected to dextran sulfate sodium (DSS)-induced IBD-like disease and then randomly divided into three groups: Model group (MD), low-dose EGCG group (LE, 20 mg/kg/d), and high-dose EGCG group (HE, 50 mg/kg/d). DSS-induced clinical and macroscopic changes were monitored daily. Intestinal permeability was assessed by FITC-Dextran assay. Results: Both high- and low-dose EGCG treatment alleviated clinical manifestations including body weight loss and disease activity index (DAI) of DSS-induced colitis. The DAI score was significantly improved after two days of EGCG treatment. At the end of the study, the macroscopic severity score (MSS) of HE and LE treatment groups were 2.4 ± 1.2, and 2.2 ± 1.0, respectively, significantly lower than that of the controls (5.0 ± 2.1). EGCG treatment also prevented colon shortening, and improved intestinal permeability and histopathological changes. In addition, EGCG treatment attenuated colon inflammation by suppressing colonic levels of pro-inflammatory cytokines IL-6, MCP-1, and TNF-alpha, and inhibited CD3+ T cell and CD68+ macrophage infiltration. Conclusion: EGCG is effective in inflammatory colitis because it reduces cellular and molecular inflammation, and reduces intestinal permeability.

## 1. Introduction

Inflammatory bowel disease (IBD) is an autoimmune disease, which involves chronic inflammation of different parts of the gastrointestinal tract resulting from an inappropriate mucosal immune response to intestinal microbes [1]. Crohn’s disease (CD) and ulcerative colitis (UC) are the two major subtypes of IBD. The incidence and prevalence of IBD is 20.2 per 100,000 person-years and 568 per 100,000 persons, respectively, in North America [2]. Both numbers have been reported to have increased over the past few decades as a result of increased urbanization, leading to an increased burden on the healthcare system [2]. Although the pathogenesis of IBD has not been fully elucidated, it is widely accepted that genetic susceptibility and environmental factors induce impairment of mucosal barrier function and translocation of intestinal microbes, which in turn, leads to the activation of both innate and adaptive immune system and production of cytokines resulting in tissue injury and clinical manifestations [1,3]. Infiltration of immune cells (such as T cells and macrophages) and increased production of pro-inflammatory cytokines (such as TNF-alpha, IL-6, IL-10) are the hallmarks during the development of IBD [1,3,4]. Current medical management includes corticosteroids, immunosuppressants, and biologic agents, several of which are sub-optimal, causing complications such as infections [1].

Experimentally induced IBD in animal models are useful tools for dissecting the pathogenesis of the disease as well as evaluating therapeutic interventions for the disease. Since its introduction in 1990, the dextran sulfate sodium (DSS)-induced rodent colitis model has become one of the most widely used animal models in IBD research due to its simplicity, reproducibility, and cost-effectiveness [5]. In this model, DSS causes erosion and increased permeability in colonic epithelium, which results in clinical signs of diarrhea, gross rectal bleeding, and weight loss [6]. Due to its close resemblance to human UC, DSS-induced colitis model has become a powerful tool in assessing novel therapeutic interventions in IBD.

Due to the lack of cure and potential side effects associated with conventional medical treatment, dietary supplements for treating IBD have been studied for decades in the hope of finding a safer and more natural therapy for IBD. Among these, green tea is one of the most widely studied and promising natural substances. Epidemiologic studies in green-tea-consuming regions have identified daily tea consumption as a protective factor in both UC and CD patients [7,8]. Green tea contains polyphenols, most of which are flavanols, a type of natural phenol and antioxidant. These polyphenols are more commonly known as catechins. Epigallocatechin gallate (EGCG), also known as epigallocatechin-3-gallate, is the most abundant catechin in green tea, responsible for many of the beneficial effects of green tea in a variety of in vitro and in vivo studies [9]. Biochemical qualities of EGCG include, but are not limited to, anti-oxidative, anti-inflammatory, and anti-carcinogenic effects [9]. In vitro studies using human intestinal or colonic epithelial cell lines have demonstrated the protective role of EGCG in intestinal inflammation through different mechanisms including down-regulation of pro-inflammatory cytokines such as IL-8, IL-6 [10,11] and inhibition of transcription factor nuclear factor-kappa B (NF-kappa B) [12]. In vivo studies using rodent colitis models have proven the effectiveness of EGCG in alleviating clinical signs of disease as well as reducing histological damage in IBD [13,14,15,16,17,18,19]. In colitis models induced by different methods, EGCG was reported to decrease a number of pro-inflammatory cytokines including interleukin-6 (IL-6) [14,17,18,19], interleukin-17 (IL-17) [18], monocyte chemoattractant protein-1 (MCP-1) [14], tumor necrosis factor-alpha (TNF-alpha) [14,17,19], and interferon-gamma (IFN-gamma) [19], while increasing anti-inflammatory cytokines like interleukin-10 (IL-10) [18] and transforming growth factor-beta (TGF-beta) [18].

## 2. Materials and Methods

### 2.1. Chemicals and Reagents

Dextran sulfate sodium (DSS) salt, MP Grade (36,000–50,000 Da), was purchased from MP Biomedicals (Santa Ana, CA). DSS solution was prepared by dissolving DSS salt in drinking water at a final concentration of 2.5% (w/v). (−)-Epigallocatechin gallate (EGCG) (≥ 95%) and fluorescein isothiocyanate conjugated dextran (FITC-dextran, 4KDa) was purchased from Sigma-Aldrich (Cat# FD4, St. Louis, MO, USA). EGCG solution was prepared by dissolving EGCG powder in phosphate buffered saline (PBS) at a final concentration of 10 mg/mL PBS and ammonium-chloride-potassium (ACK) lysing buffer was purchased from Thermo Fisher Scientific (Waltham, MA, USA).

### 2.2. Animal Care and Experiment Design

Male C57BL/6J mice (8 weeks old, body weight 22–30 g) were purchased from The Jackson Laboratory (Bar Harbor, ME). All mice were housed in a pathogen-free animal facility and maintained at 22 ± 2 °C and on a 12-hour light-dark cycle condition. Ad libitum supply of standard rodent chow and drinking water were provided. The animal experiments were conducted in accordance with the Guide for the Care and Use of Laboratory Animals (NAP 2011) and the animal use protocol was approved by the Institutional Animal Care and Use Committee at the University of Houston.

**Figure 1:**
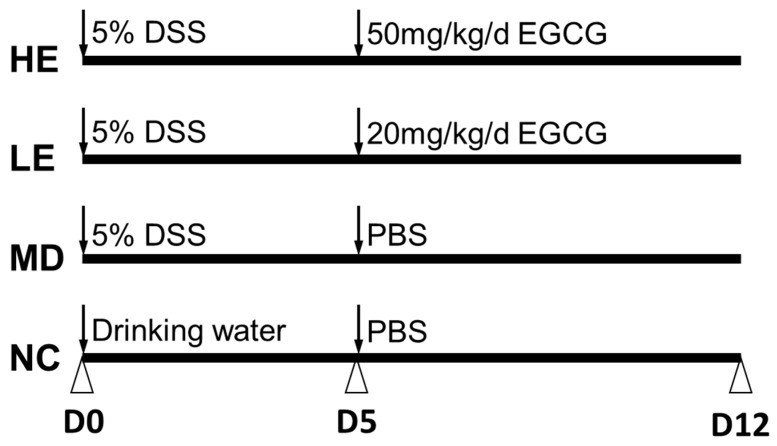
Epigallocatechin gallate (EGCG) treatment schedule for the study. Mice were randomly divided into four groups based on body weight. Normal control group mice (NC) were given normal drinking water from Day 0 to Day 5 followed by oral gavage with phosphate buffered saline (PBS) from Day 5 to Day 12. Dextran sulfate sodium (DSS)-induced colitis model group mice (MD) were administered 2.5% DSS containing drinking water from Day 0 to Day 5 followed by oral gavage with PBS from Day 5 to Day 12. Low-dose (LE) and high-dose (HE) EGCG treatment groups of mice were given 2.5% DSS containing drinking water from Day 0 to Day 5 followed by oral gavage with 20 mg/kg/d and 50 mg/kg/d EGCG, respectively, from Day 5 to Day 12.

Colitis was induced by 2.5% (w/v) DSS in drinking water. The mice were randomly divided into four groups (*n* = 8 mice per group): Normal control group where disease was not induced (NC), a model control group where disease was induced but no EGCG was administered (MD), a low-dose (20 mg/kg/d) EGCG group (LE), and a high-dose (50 mg/kg/d) EGCG group (HE), following induction of colitis (Figure 1).

Mice in NC were given ad libitum access to drinking water for 5 days followed by oral gavage with PBS for 7 days. Mice in MD were given 2.5% DSS containing drinking water for 5 days followed by oral gavage with PBS for 7 days. Mice in LE and HE groups were given 2.5% DSS-containing drinking water for 5 days followed by oral gavage with 20 mg/kg/d EGCG for LE and 50 mg/kg/d EGCG for HE for 7 days (10 mg/mL drinking fluid, Sigma-Aldrich, St. Louis, MO; USA).

### 2.3. Disease Monitoring and Sample Collection

All mice were evaluated and weighed daily by an unbiased observer. Water consumption was measured after 5 days for each mouse. Stool consistency and blood in the feces were evaluated daily using fresh stool. Occult blood was tested using the Hemoccult II^®^ test (Beckman Coulter, Brea, CA, USA). Disease activity index (DAI) was determined daily by totaling the scores for weight loss (0: <5%, 1: 5%–10%, 2: 10%–20%, 4: >20%), stool consistency (0: Normal, 2: Loose stools, 4: Watery diarrhea), and bleeding (0: Negative, 1: Hemoccult positive, 2: Hemoccult positive and visual pellet bleeding, 4: Gross bleeding), as described previously [20]. Macroscopic severity score (MSS) was assessed terminally by summating the total score for rectal bleeding (1: None, 2: Red, 3: Dark red, 4: Gross bleeding), rectal prolapse (0: No, 1: Signs of prolapse, 2: Clear prolapse, 3: Extensive prolapse), stool consistency (0: Normal, 1: Soft, 2: Very soft, 3: Diarrhea), and color (1: Normal, 2: Red, 3: Dark red, 4: Black), as previously described [20].

At the end of the study (Day 12), all mice were euthanized by carbon dioxide inhalation followed by cervical dislocation. Spleens and pericolic mesenteric lymph nodes were removed and placed in ice-cold PBS for flow cytometry analysis. The entire colorectum (from colocecal junction to the anal verge) was removed and the length was measured. Feces inside the colon were removed carefully by rinsing with PBS using a gavage needle. The colon was divided equally into three parts: Proximal, middle, and distal. The middle part was placed in 10% formalin solution immediately for pathological assessment. The proximal and distal parts were weighed and frozen at −70 °C for myeloperoxidase (MPO) activity testing and cytokine assays, respectively. Mesenteric lymph nodes were removed and placed in ice-cold PBS for flow cytometry analysis.

### 2.4. Histopathological Assessment

For pathological assessment, the middle section of the colon tissue was placed in a tissue cassette and submerged in 10% formalin. Then, 5 μm paraffin-embedded cross sections were prepared and stained with hematoxylin and eosin (H & E). The H & E stained sections were evaluated by a blinded pathologist using two different scoring systems. The IBD score (maximum = 11) was the sum of the following measures as described previously [21]: Crypt architecture (0: Normal, 3: Severe crypt distortion with loss of entire crypts), degree of inflammatory cell infiltration (0: Normal, 3: Dense inflammatory infiltrate), muscle thickening (0: Base of crypt sits on the muscularis mucosae, 3: Marked muscle thickening present), goblet cell depletion (0: Absent, 1: Present), and crypt abscess (0: Absent, 1: Present). The colitis score (maximum = 8) was the sum of the following two features [22]: Epithelium (0: Normal morphology; 1: Loss of goblet cells, 2: Loss of goblet cells in large areas; 3: Loss of crypts; 4: Loss of crypts in large areas), and infiltration (0: No infiltrate; 1: Infiltrate around crypt basis; 2: Infiltrate reaching to lamina muscularis mucosae; 3: Extensive infiltration reaching the lamina muscularis mucosae and thickening of the mucosa with abundant edema; 4: Infiltration of the lamina submucosa).

### 2.5. Immunofluorescence Staining

The middle section of the colon tissue was fixed in 10% formalin overnight and 5 μm paraffin embedded cross sections were prepared. After dewaxing and rehydration, tissue sections were boiled for 20 min in 10 mmol/L citrate buffer (pH 6.0). The sections were blocked with blocking buffer for one hour and then incubated with anti-mouse CD68 mAb (for macrophages, 1:100, Abcam Inc, Cambridge, MA, USA, ab955) or rat anti-mouse CD3 (for T cells, 1:50, Abcam Inc, Cambridge, MA, USA, ab11089) mAb separately at 4 °C overnight. After washing, the sections were incubated with Alexa Fluor^®^ 555 conjugated goat anti-mouse IgG (H+L) (for macrophages, 1:1000, Thermo Fisher Scientific Inc, Waltham, MA, USA, A32727) or Alexa Fluor^®^ 488 conjugated goat anti-rat IgG (H+L) (for T cells, 1:1000, Thermo Fisher Scientific Inc, Waltham, MA, USA, A-11006) for two hours at room temperature. The sections were then counterstained and mounted with ProLong^®^ Gold Antifade Mountant with DAPI (Thermo Fisher Scientific Inc, Waltham, MA, USA, P36931). Rabbit isotype control IgG (Abcam Inc, Cambridge, MA, USA, ab27478) at the same dilution was used as control. For each slide, 10 randomly chosen fields (20X) were examined and counted for fluorescent cells. The result for each slide was expressed as the average cell counts in ten fields.

### 2.6. Intestinal Permeability Analysis

Intestinal permeability was assessed using the FITC-dextran permeability assay. On Day 12, mice were starved from water overnight but allowed free access to food. The next morning, mice were fed with FITC-dextran (44 mg per 100 g body weight) by oral gavage. After 4 h, whole blood was collected, and serum was isolated. Serum was diluted with equal volume of PBS and tested in duplicate. The concentration of FITC-dextran was determined using a fluorometer with dilutions of FITC-dextran in PBS as a standard curve.

### 2.7. Myeloperoxidase (MPO) Immunoassay

Colonic MPO levels reflect the degree of neutrophil infiltration. MPO levels in the distal part of the colonic tissue were measured using a sandwich enzyme-linked immunosorbent assay (ELISA). Briefly, approximately 50–100 mg thawed colonic tissue was homogenized on ice in PBS. The homogenate was centrifuged at 1500 rpm for 6 min at 4 °C and the supernatant was collected. The supernatant was used for protein analysis with a BCA protein assay kit (Thermo Scientific, Waltham, MA, USA) and for MPO immunoassay. MPO in the supernatant was assayed using commercially available mouse MPO ELISA kit (Cat# DY3667, R&D Systems, Minneapolis, MN, USA) according to the manufacturer’s manual. MPO levels were then normalized by total protein concentration and expressed as pg/mg protein.

### 2.8. Cytokine Immunoassay

The distal part of the colonic tissue was homogenized similar to the proximal part. Cytokine (IL-6, IL-10, MCP-1, and TNFα) levels were measured using commercially available ELISA kits (Cat# DY406, DY417, DY479, DY410, R&D Systems, Minneapolis, MN, USA) according to the manufacturer’s manual. Cytokine levels were normalized by tissue weight and expressed as ng/g tissue.

### 2.9. Statistical Analysis

All data were expressed as mean ± SD. Comparison between two groups was performed using the Student’s t test. One-way analysis of variance (ANOVA) followed by Tukey’s multiple comparison test were used for the comparison of three or more groups. A 2-tailed *P* value < 0.05 was considered statistically significant. All statistical analyses were performed using GraphPad Prism (V.6.0, GraphPad, San Diego, CA, USA).

## 3. Results

### 3.1. EGCG Treatment Alleviated DSS-induced Clinical and Macroscopic Changes

**Figure 2:**
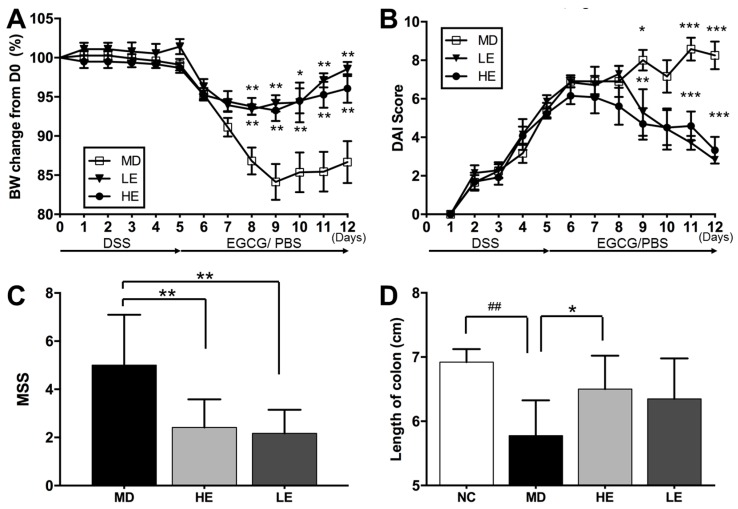
Effect of EGCG treatment on body weight, disease activity index (DAI) score, macroscopic severity score, and colon length following DSS-induced colitis. Both high and low doses of EGCG treatment significantly improved DSS-induced (**A**) body weight loss, (**B**) DAI score, (**C**) macroscopic severity score (MSS). High-dose EGCG markedly restored (**D**) colon length. Body weight changes were measured daily and calculated as the percentage change from Day 0. DAI scores were assessed from Day 1 to Day 12. MSS and colon length were evaluated at the end of the study. BW: Body weight, DAI: Disease activity index, DSS: Dextran sulfate sodium, EGCG: Epigallocatechin gallate, MSS: Macroscopic severity score, PBS: Phosphate buffered saline; NC: Normal control group, MD: Colitis model group, HE: High-dose EGCG group, LE: Low-dose EGCG group. Data are presented as mean ± standard deviation. * *p* < 0.05 vs. MD, ** *p* < 0.01 vs. MD, *** *p* < 0.001 vs. MD, ## *p* < 0.01 vs. NC.

As shown in Figure 2, both high and low doses of EGCG treatment were able to alleviate clinical manifestation of DSS-induced colitis in mice, including body weight loss and DAI.

In the HE and LE groups, body weight loss was significantly improved from Day 8 (Figure 2A). The DAI disease score was significantly reduced on Day 9, Day 11, and Day 12 (Figure 2B). At the end of the study, DAI was 3.3 ± 2.4 and 2.8 ± 0.4 in HE and LE groups, respectively (as compared to 8.3 ± 2.5 in MD group, *p* < 0.001). EGCG also significantly reduced the MSS disease score in both the high-dose (2.4 ± 1.2, *p* = 0.001) and low-dose EGCG groups (2.2 ± 1.0, *p* = 0.003) compared to the MD group (5.0 ± 2.1) (Figure 2C). DSS-induced colitis caused a shortening of colon length. When compared to normal untreated mice (NC group: 6.9 ± 0.2 cm), the colon length of DSS-treated mice (MD group: 5.8 ± 0.5 cm) was significantly shortened (*p* = 0.002). Treatment with EGCG restored colon length (HE group: 6.5 ± 0.5 cm, *p* = 0.015 vs. MD; LE group: 6.4 ± 0.6 cm, *p* = 0.136 vs. MD) (Figure 2D).

### 3.2. EGCG Treatment Improved Intestinal Histopathological Changes

**Figure 3:**
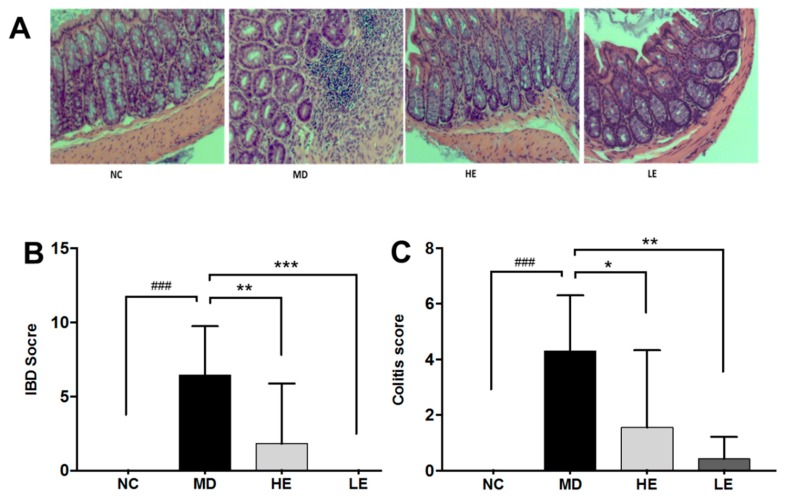
Effect of EGCG treatment on histopathologic changes in DSS-induced colitis. Hematoxylin and eosin (H & E) stained images of representative histopathologic changes in the four groups of mice are shown (**A**). Two different histopathologic scores were used to evaluate the histopathologic changes, namely inflammatory bowel disease (IBD) score (**B**) and colitis score (**C**). Both high- and low-dose EGCG treatment significantly decreased both the IBD score and the colitis score. H & E: Hematoxylin and eosin, IBD: Inflammatory bowel disease, NC: Normal control group, MD: Colitis model group, HE: High-dose EGCG group, LE: Low-dose EGCG group. Data are presented as mean ± standard deviation. * *p* < 0.05 vs. MD, ** *p* < 0.01 vs. MD, *** *p* < 0.001 vs. MD, ### *p* < 0.001 vs. NC.

As shown in Figure 3A, DSS-induced colitic mice showed significant histopathological changes in the colon, including acute inflammation characterized by epithelial erosion and ulceration, crypt abscess, loss of goblet cells, mucosal erosion and edema, and substantial neutrophil infiltration.

The current study used both the IBD score and colitis score to evaluate histopathological changes in the colon, as previously described [21,22]. Both the IBD score and the colitis score showed significant alleviation of histopathological injury in colon by both low and high dose of EGCG treatment. As displayed in Figure 3B, high-dose EGCG decreased IBD pathologic score from 6.4 (range 2–11) to 1.8 (range 0–10, *p* = 0.005 vs. MD), while low-dose EGCG decreased the score to 0 (range 0–0, *p* < 0.001 vs. MD). As displayed in Figure 3C, high-dose EGCG decreased the colitis score from 4.3 (range 1–7) to 1.6 (range 0–7, *p* = 0.01 vs. MD), while low-dose EGCG decreased the score to 0.4 (range 0–2, *p* = 0.001 vs. MD).

### 3.3. EGCG Treatment Improved Epithelial Barrier Integrity

**Figure 4:**
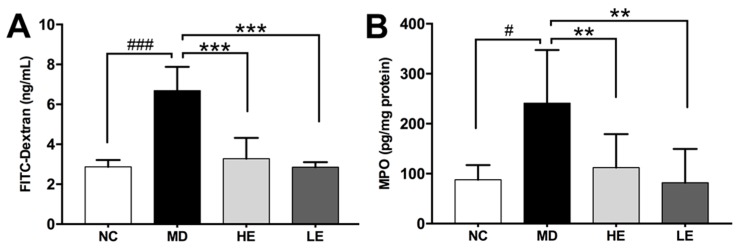
Effect of EGCG treatment on intestinal permeability (**A**), and myeloperoxidase (MPO) levels (**B**) in DSS-induced colitis. Intestinal permeability was measured using the fluorescein isothiocyanate conjugated dextran (FITC-dextran) assay on Day 12. At the end of the study, the colon was harvested and divided equally into three parts. MPO levels in the distal part of the colonic tissue was measured as a marker of neutrophil infiltration. DSS treatment significantly increased serum FITC-dextran levels and colon tissue MPO levels. Both high-and low-dose EGCG treatment decreased serum FITC-dextran levels and colonic tissue MPO levels significantly. FITC-dextran: Fluorescein isothiocyanate conjugated dextran, MPO: Myeloperoxidase; NC: Normal control group, MD: Colitis model group, HE: High-dose EGCG group, LE: Low-dose EGCG group. Data are presented as mean ± standard deviation. ** *p* < 0.01 vs. MD, *** *p* < 0.001 vs. MD, # *p* < 0.05 vs. NC, ### *p* < 0.001 vs. NC.

DSS-induced colitis is accompanied by increased intestinal permeability as assessed by the FITC-Dextran assay. As shown in Figure 4A, mice with DSS-induced colitis (MD group: 6.69 ± 1.19 ng/mL, *p* < 0.001 vs. NC) exhibited significantly increased serum level of FITC-Dextran compared to normal untreated mice (NC group: 2.87 ± 0.34 ng/mL). Both low- and high-dose EGCG improved intestinal permeability significantly (HE group: 3.28 ± 1.00 ng/mL, *p* < 0.001 vs. MD; LE group: 2.85 ± 0.25ng/mL, *p* < 0.001 vs. MD) (Figure 4A).

### 3.4. EGCG Treatment Inhibited Inflammation in Colonic Tissue

In order to explore the mechanisms underlying the therapeutic effects of EGCG in DSS-induced colitis, we focused on mediators of inflammation in the colonic tissue. First, elevated MPO was observed in DSS-induced colitic mice (MD) compared to normal untreated mice (NC) (241.2 ± 106.4 vs. 87.76 ± 29.47 pg/mg protein, *p* = 0.014). Importantly, EGCG treatment markedly reduced MPO levels, a marker of neutrophil infiltration, to 47% in the HE group (112.2 ± 66.82 pg/mg protein, *p* = 0.008) and 34% in the LE group (81.7 ± 67.93 pg/mg protein, *p* = 0.005) (Figure 4B).

**Figure 5:**
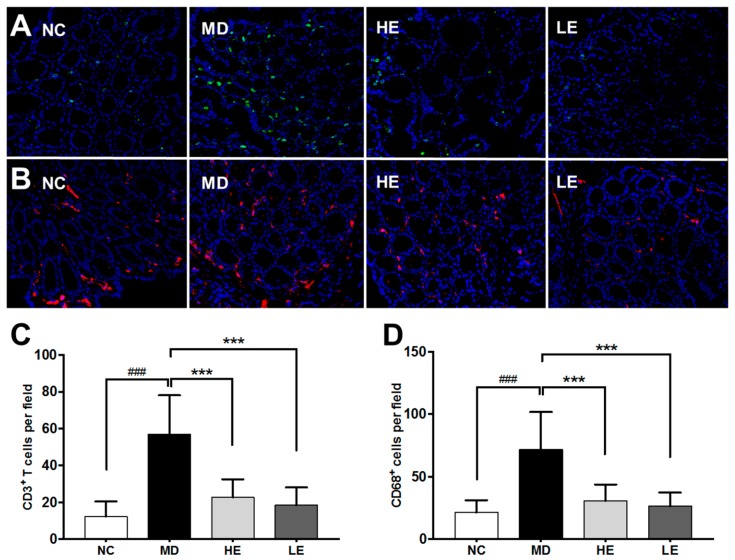
The effect of EGCG treatment on immune cell infiltration in colonic tissue in DSS-induced colitis. Representative immunofluorescence staining for CD3+ T cells (**A**) and CD68+ macrophages (**B**) in the NC, MD, HE, and LE groups of mice (from left to right, 40x) are displayed. Ten randomly chosen fields (20X) per section were examined for fluorescent cells in each section and average cell numbers per field are shown. (**C**,**D**) show the quantitation of the same data; DSS-induced colitic mice exhibited significantly increased infiltration of T cells and macrophages in the colonic tissue. Both high-dose and low-dose EGCG treatment were able to decrease the number of infiltrating T cells (**C**) and macrophages (**D**) significantly when compared to the non-treated MD group. NC: Normal control group, MD: Colitis model group, HE: High-dose EGCG group, LE: Low-dose EGCG group. Data are presented as mean ± standard deviation. *** *p* < 0.001 vs. MD; ### *p* < 0.001 vs. NC.

Second, immunofluorescence staining using anti-CD3 and anti-CD68 was applied to evaluate T cell and macrophage infiltration, respectively (Figure 5A,B).

In normal control mice, only few T cells and macrophages were detectable in the colonic tissue. However, in DSS-induced colitis mice, significantly increased infiltration of T cells (*p* < 0.001, Figure 5C) and macrophages (*p* < 0.001, Figure 5D) were observed. Both high-dose and low-dose EGCG treatment decreased the number of infiltrating T cells and macrophages significantly as compared to non-treated MD group (Figure 5C,D).

**Figure 6:**
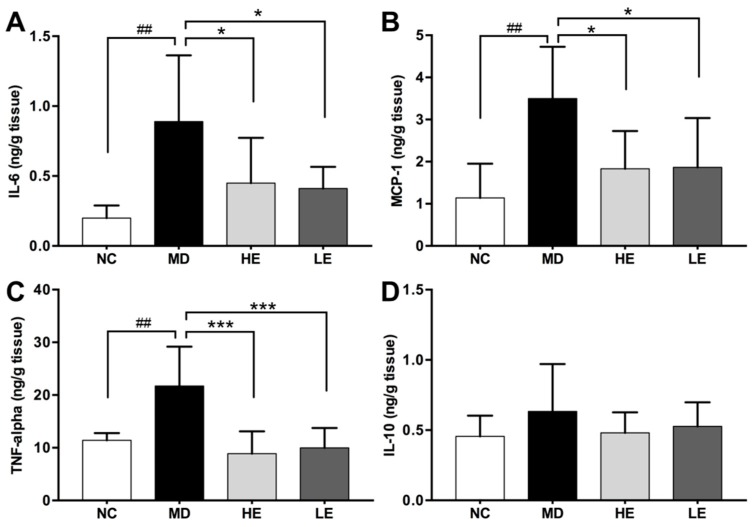
Effect of EGCG treatment on colonic tissue levels of inflammatory cytokines in DSS-induced colitis. At the end of the study, the colon was harvested and divided equally into three parts. Cytokine levels in homogenate of the proximal part of the colonic tissue were measured by ELISA. Pro-inflammatory cytokines IL-6 (**A**), MCP-1 (**B**), TNF-alpha (**C**) levels in the colonic tissue were significantly increased in DSS-induced colitis model while treatment with both high and low dose of EGCG significantly decreased them. No significant differences were observed in the colonic tissue levels of IL-10 (**D**). IL-6: Interleukin 6, MCP-1: Monocyte chemoattractant protein-1, TNF-alpha: Tumor necrosis factor alpha, NC: Normal control group, MD: Colitis model group, HE: High-dose EGCG group, LE: Low-dose EGCG group. Data are presented as mean ± standard deviation. * *p* < 0.05 vs. MD, *** *p* < 0.001 vs. MD, ## *p* < 0.01 vs. NC.

Third, DSS-induced colitic mice (MD) exhibited significantly increased colonic tissue levels of pro-inflammatory cytokines compared to normal untreated mice (NC), which included IL-6 (0.89 ± 0.47 vs. 0.2 ± 0.09 ng/g tissue, *p* = 0.004), MCP-1 (3.5 ± 1.23 vs. 1.14 ± 0.81 ng/g tissue, *p* = 0.007), and TNF-alpha (21.7 ± 7.47 vs. 11.41 ± 1.38 ng/g tissue, *p* = 0.006) (Figure 6).

Treatment with EGCG inhibited the elevation of IL-6 (HE group: 0.45 ± 0.32 ng/g tissue, *p* = 0.03 vs. MD; LE group: 0.41 ± 0.15 ng/g tissue, *p* = 0.03 vs. MD, Figure 6A), MCP-1 (HE group: 1.83 ± 0.89 ng/g tissue, *p* = 0.01 vs. MD; LE group: 1.87 ± 1.17 ng/g tissue, *p* = 0.01 vs. MD, Figure 6B), and TNF-alpha (HE group: 8.87 ± 4.24 ng/g tissue, *p* < 0.001 vs. MD; LE group: 10 ± 3.76 ng/g tissue, *p* < 0.001 vs. MD, Figure 6C) in both low- and high-dose EGCG groups. However, no significant difference was observed in the colonic tissue level of anti-inflammatory cytokine IL-10 among the four groups of mice (Figure 6D).

## 4. Discussion

Green tea and its major polyphenolic component EGCG have been shown to be beneficial in ulcerative colitis both in clinical trials [23] and in vivo animal studies [13,14,15,16,17,18,19]. The underlying mechanism of action has not been fully elucidated. In this study, we demonstrated the efficacy of two different doses of EGCG in treating DSS-induced acute colitis, and the underlying mechanism of action including the suppression of inflammation in the colonic tissue. The results of this study expand upon previous studies on the therapeutic effects of EGCG in DSS-induced colitis model using a different animal background and different treatment dosage, which may be of clinical importance since green tea may provide a safer and more natural therapy for IBD.

In the present study, both the 20 mg/kg/d and 50 mg/kg/d EGCG treatment regimens attenuated DSS induced weight loss as well as clinical symptoms as reflected by the DAI score. Although no significant differences were observed between the two dosage groups, the lower dosage group tended to have higher body weight and lower DAI scores at the end of the study (Day 11 and Day 12), indicating a preferred dosage of 20 mg/kg/d in treating DSS-induced colitis in C57BL/6J mice. Although C57BL/6J mice have been recommended as a DSS-induced colitis model [5], due to the chronic nature of the disease mimicking human IBD, it has never been utilized in EGCG treatment studies. Our study, thus, confirms the previous studies on DSS-induced colitis using mice of a different background; moreover, the dosage differences in this study further complement results from previous studies [14,18]. Xu et al. [18] used 50 mg/kg/d and 100 mg/kg/d EGCG to treat DSS-treated BALB/c mice and demonstrated a dose-dependent effect on reducing DAI scores. Bitzer et al. [14] used 3.2 mg/mL EGCG-containing drinking water, which equates to 480 mg/kg/d (assuming mice drink 15 mL/100 g body weight of water per day), to treat DSS-treated CF-1 mice. At this high dosage, EGCG was found to significantly accelerate DSS-induced body weight loss. Guan et al. [24] fed DSS-treated CD-1 mice a diet containing 0.1% to 0.5% EGCG, which equates to 150 mg/kg/d to 750 mg/kg/d (assuming mice consume 15 g of food per day per 100 g of body weight). Here, the higher dose of EGCG exacerbated bleeding and body weight loss. The adverse effects of the higher dose of EGCG reported in the latter study contrast with the better efficacy results observed using the lower dosages of EGCG used in the present study. The underlying mechanistic basis of the toxicity reported with higher doses of EGCG that others have reported warrants further investigation.

Pathological changes in DSS-induced colitis at a macroscopic level consist of a shortened edematous colon with hemorrhage and ulceration. Histologically, edematous mucosa with infiltration of neutrophils and lymphocytes is seen [25], closely resembling human IBD, particularly ulcerative colitis. We demonstrated a significantly increased histological damage score and shortening of the colon after DSS administration, while EGCG treatment alleviated the macroscopic and histological changes in the colon. In evaluating the histopathologic changes in the colonic tissue, we adopted both the IBD score and colitis score. Both scoring systems underscored the beneficial effect of EGCG in improving the histopathologic changes associated with IBD. Moreover, the pathological changes in the diseased colon functionally compromised the intestinal barrier, causing increased intestinal permeability, which could be the inciting trigger for the inflammatory responses in IBD [1]. EGCG treatment decreased serum levels of FITC-dextran compared to DSS only treatment, indicating the ability of EGCG in normalizing intestinal permeability and protecting intestinal epithelial barrier function in IBD, which is in accordance with a previous study using a different in vivo assay for intestinal permeability [14].

Since EGCG has been ascribed anti-inflammatory properties in several other autoimmune diseases [26,27], we examined whether EGCG protects against IBD through its anti-inflammatory potential. Importantly, EGCG treatment significantly decreased immune cell infiltration in colonic tissue as measured by colonic MPO levels and immunofluorescence staining for infiltrating immune cells when compared to the controls. EGCG has been reported to inhibit MPO activity both in vitro and in vivo in various other disease models [28,29,30,31]. MPO is a peroxidase excessively expressed in neutrophils, which catalyzes the production of reactive oxygen species (ROS). The inhibitory effect of EGCG on MPO could potentially lead to reduced production of ROS, in line with the well-established role of EGCG as an antioxidant [26,32].

Aberrant cytokine production by immune cells plays a pivotal role in regulating intestinal inflammation and associated manifestations in IBD [1,3]. Indeed, we found increased levels of pro-inflammatory cytokines IL-6, MCP-1, and TNF-alpha in colonic tissue of colitic mice and a normalization effect of EGCG on these cytokines, in resonance with results from previous studies of EGCG using different colitis models [14,17,18,19]. However, we did not observe any significant alterations in colonic tissue levels of IL-10, an anti-inflammatory cytokine that acts on both the innate and adaptive arms of the immune system. Human genetic studies have documented the association of single-nucleotide polymorphisms (SNPs) in *IL10* with IBD [33,34]. Animal studies have shown that IL-10-deficient mice developed chronic enterocolitis spontaneously, where treatment with low- to medium-dose EGCG alleviated symptoms [17,35]. Although circulating IL-10 was reported to be elevated both in animal models of IBD and in human studies [13,17,36], intestinal epithelial cells from healthy and inflamed colonic tissue expressed similar levels of IL-10 mRNA and protein [37]. Moreover, IL-10 treatment for IBD has not been successful as anticipated [37]. All the above suggest that the contributions of IL-10 to IBD pathogenesis is complex and multi-factorial, warranting further study.

In summary, the results of the present study re-affirm that EGCG alleviates clinical symptoms, restores intestinal permeability, and mitigates colonic inflammation in the DSS-induced murine IBD model. The therapeutic effect may be due to the suppression of pro-inflammatory cytokine production in the colonic tissue. These findings strengthen existing evidence supporting the use of EGCG or EGCG-containing natural ingredients in the treatment of IBD. Several issues need to be addressed in order to translate the results from this study to the clinics. First, the DSS-induced colitis model used in this study is an acute disease model, while IBD in patients is a chronic relapsing disease. It is not clear if EGCG would be equally protective against chronic intestinal inflammation. Second, the dose-dependent effect of EGCG should be further investigated in humans since the dosages of EGCG used in murine studies may not translate well to humans.

## Abbreviations

IBD	Inflammatory bowel disease
EGCG	Epigallocatechin gallate
DSS	Dextran sulfate sodium
CD	Crohn’s disease
UC	Ulcerative colitis
IL-6	Interleukin-6
MCP-1	Monocyte chemoattractant protein-1
IL-10	Interleukin-10
TNF-α	Tumor necrosis factor-alpha
DAI	Disease activity index
MSS	Macroscopic severity score
MPO	Myeloperoxidase
